# Gaps in evidence for the use of medically authorized cannabis: Ontario and Alberta, Canada

**DOI:** 10.1186/s12954-021-00509-0

**Published:** 2021-06-08

**Authors:** Cerina Lee, Jessica M. Round, Scott Klarenbach, John G. Hanlon, Elaine Hyshka, Jason R. B. Dyck, Dean T. Eurich

**Affiliations:** 1 School of Public Health, University of Alberta, 2-040 Li Ka Shing Centre for Health Research Innovation, 11203-87 Avenue, Edmonton, AB T6G 2E1 Canada; 2 St. Michael’s Hospital Department of Anesthesia, University of Toronto, Toronto, ON Canada; 3 Department of Anesthesiology and Pain Medicine, University of Toronto, Toronto, ON Canada; 4 Cardiovascular Research Centre, Department of Pediatrics, Faculty of Medicine and Dentistry, University of Alberta, Edmonton, AB Canada; 5 Division of Nephrology, Faculty of Medicine and Dentistry, University of Alberta, Edmonton, AB Canada

**Keywords:** Medical cannabis, Epidemiology, Cohort study, Chronic pain, Anxiety, Depression, Cancer, Spasticity, Nausea

## Abstract

**Background:**

With legal access to medical cannabis in Canada since 2001, there is a need to fully characterize its use at both the individual and population levels. We draw on data from Canada’s largest cohort study of medical cannabis to identify the primary reasons for medical cannabis authorization in Canada from 2014 to 2019 in two major provinces: Alberta (AB) and Ontario (ON), and review the extent that evidence supports each indication.

**Methods:**

Self-reported baseline assessments were collected from adult patients in ON (*n* = 61,835) and AB (*n* = 3410) who were authorized medical cannabis. At baseline, sociodemographic, primary medical information, and validated clinical questionnaires were completed by patients as part of an individual assessment. Patients’ reasons for seeking medical cannabis were compared to published reviews and guidelines to assess the level of evidence supporting medical cannabis use for each condition.

**Results:**

Medical cannabis use in both AB and ON was similar in both demographic and reason for authorization. The most common reasons for medical cannabis authorization were: (1) pain (AB = 77%, ON = 76%) primarily due to chronic musculoskeletal, arthritic, and neuropathic pain, (2) mental health concerns (AB = 32.9%, ON = 38.7%) due to anxiety and depression, and (3) sleep problems (AB = 28%, ON = 25%). More than 50 other conditions were identified as reasons for obtaining authorization.

**Conclusion:**

In both AB and ON, the majority of reasons for medical cannabis authorization are not substantiated by clinical evidence to fully support its efficacy for long-term use. Ongoing epidemiological studies on medical cannabis on these treatments are warranted to fully outline its treatment benefits or risks.

**Supplementary Information:**

The online version contains supplementary material available at 10.1186/s12954-021-00509-0.

## Introduction

The legal access of medical cannabis has been available to patients as a potential therapeutic avenue of treatment for nearly a decade in Canada [1]. Although legally available, there is an absence of randomized controlled trials and high-quality longitudinal cohort studies that support cannabis as a proven medical therapy [2]. This evidence gap persists in the medical cannabis research field and hinders clinicians and physicians ability to provide evidence-based healthcare to patients seeking medical cannabis for a wide spectrum of therapeutic needs [3]. With the recent legalization of non-medical cannabis (October 2018) in Canada, there has been a growing public interest in the therapeutic use of cannabis. Thus, medical evidence is needed to support its use [4]. Consequently, this will enable physicians to make clinically informed healthcare decisions surrounding medical cannabis authorization [5, 6].

To date, systematic reviews and clinical studies have reported a wide spectrum of underlying reasons for medical cannabis prescription—with mixed outcomes [7, 8] on both the types of reasons for use and evidence on efficacy for its therapeutic effects. The majority of clinical studies most frequently report the following reasons for its authorization: (1) chronic pain [9, 10] (back pain, neuropathic pain, arthritic pain, pain from non-cancer [11] and cancer [9], etc.); (2) mental health conditions [12–15] (anxiety [16, 17], depression [18, 19]); (3) autoimmune disorders [20]; (4) sleep problems [21, 22]; (5) neurological [23]; (6) gastrointestinal [24]; and (7) other health conditions [25, 26] such as chemo-induced nausea/vomiting [7]. There also has been an increasing frequency of medical cannabis use as an alternative to opioids [11, 18, 27] for patients. With the exception of neuropathic chronic pain [10], chemotherapy-induced nausea/vomiting [28], and certain spasticity symptoms, the majority of previous studies concur that there is currently very weak evidence regarding its clinical effectiveness for its long-term use [23]. Furthermore, the studies to date are predominantly limited to small cohort sample sizes [11], very few are conducted within Canada [4], and the majority do not differentiate between medical and non-medical cannabis usage [29]. Duration of trials and limitations in blinding are also significant limitations.

Currently, two guidelines guide medical cannabis authorizers and outline the best-available evidence (clinical trials, epidemiological studies, and systematic reviews) [5, 30]. In both guidelines, clinicians report caution when utilizing cannabis as a first- or second-line treatment for any type of health condition. Despite substantive evidence for the therapeutic effects of medical cannabis on chronic pain (cancer, arthritic, neurologic, musculoskeletal pain), there is insufficient or limited evidence for medical cannabis use on the majority of other health conditions [30]. Yet, continued use of medical cannabis as a treatment suggests the critical need for ongoing surveillance and monitoring of medical cannabis authorization, which can inform clinicians about the therapeutic needs of current patients. Moreover, ongoing surveillance can identify conditions for which patients are seeking medical cannabis which will assist researchers in closing the evidence gaps for medical cannabis within these conditions. Thus, our study aims to provide this new and relevant demographic and clinical data from one of the largest databases of patients seeking cannabis as a medical therapy by describing the primary reasons for medically authorized cannabis use in a large cohort of patients in Ontario (ON) and Alberta (AB), Canada, and to descriptively compare these conditions with current recommendations for use of medical cannabis in Canada and the USA.

## Methods

### Study design and population

A cohort study was conducted of all adult patients authorized to access medical cannabis [inhaled (smoked or vaporized) or orally consumed cannabis] attending a chain of specialized clinics in AB and ON, Canada between April 2014 and January 2019. Our study includes individuals at least 18 years of age, of any sex and ethnicity, who received medical cannabis authorization for any indication (acute and chronic). Patients may choose to seek assessment for medical cannabis through the clinic via a self-referral or by a physician referral.

### Data source

Informed written consent was provided by the patient at intake, which allowed data to be collected and used for clinical and research purposes. As part of the intake process, each patient seeking medical cannabis met with a counselor who performed an initial assessment and collected relevant data. All patients must provide sociodemographic information and disclose their primary medical complaints that constitute their rationale for requesting medical cannabis authorization. Many patients completed self-reported assessments to assist physicians in determining whether the patient was a suitable candidate for medical cannabis. These assessments included: the Generalized Anxiety Disorder 7-item (GAD-7) scale; Patient Health Questionnaire (PHQ-9); and the CAGE Questionnaire Adapted to Include Drugs (CAGE-AID). Following their initial intake interview, the patient would then be referred to a physician who makes their assessment based on the self-reported information, the patient’s health record, and any additional relevant health information.

### Patient and public involvement

Patients and the public were not involved in designing, conducting, or reporting this research project as it was not directly applicable to this project.

## Ethics approval

The study was approved by the University of Alberta Health Research Ethics Board (PRO 00068887) and the Veritas Research Ethics Board in Ontario (16111-13:21:103-01-2017).

### Descriptive analyses

For each province, sociodemographic characteristics including age at authorization, sex, neighborhood average income quintile, GAD-7, PHQ-9, and CAGE-AID score, and reason for cannabis use were analyzed descriptively using counts and percentages. Neighborhood average income was determined by matching census data to the current area of residence for the patient. The neighborhood average income for each province was split into quintiles with quintile 1 representing the lowest income and quintile 5 the highest income. The GAD-7 was used to assess generalized anxiety disorder, PHQ-9 was used to assess depression, and CAGE-AID was used to assess problems with drugs or alcohol. Reason for cannabis use was categorized based on keywords around the cannabis indication in the physician’s notes (Additional file 1: Table S1). Patients reporting multiple reasons for seeking medical cannabis were coded into multiple categories. Patients whose records did not identify their reasons for seeking medical cannabis or the reason was very infrequent were coded as having an “uncategorized” reason for seeking medical cannabis.

Patient’s reasons for seeking cannabis were coded into 52 different categories (Additional file 1: Table S1). Eight of these 52 categories were considered high-level categories into which the other categories were grouped. These high-level categories include pain, mental health, autoimmune conditions, sleep problems, neurological conditions, gastrointestinal conditions, other, and uncategorized.

### Evidence appraisal protocol

The evidence to support cannabis use for each category reason was examined using published reviews and guidelines. A formal search was conducted for all available reviews and/or guidelines using PubMed. Timeline was restricted to the most recent 5 years (2015–2020) and the search was restricted to systematic reviews conducted in English, literature reviews and scoping reviews. A separate full systematic or literature review was not needed for this study as we are simply comparing our findings to pre-existing and current literature reviews available on cannabis. As seen in Additional file 1: Appendix A, we identified a total of 41 systematic and literature reviews. For each category and subcategory, a search was conducted on PubMed for an existing systematic or literature review specific to each category within the past 5 years. Two reviewers determined if there was any evidence and if applicable the level of that evidence.

### ***Guiding framework for medical cannabis ***[30]

The numbering system to assess the level of evidence was directly referenced from the National Academies of Science Engineering and Medicine’s (NASEM) recommendation standards for medical cannabis. We used the same numerical and categorical levels of evidence as defined by NASEM:

Conclusive or substantial evidence (3): There is strong evidence from randomized controlled trials to support the conclusion that cannabis or cannabinoids are an effective or ineffective treatment for the health endpoint of interest.

Moderate or limited evidence (2): There is some evidence to support the conclusion that cannabis or cannabinoids are an effective or ineffective treatment for the health endpoint of interest.

Weak evidence or insufficient (1): There is weak or insufficient/no evidence to support the conclusion that cannabis or cannabinoids are an effective or ineffective treatment for the health endpoint of interest.

As noted by the level of evidence in the reviews or guidelines, we assigned a number for each category: 1 (insufficient or weak evidence), 2 (limited or moderate evidence), or 3 (substantial or conclusive evidence). If there were no existing reviews, we left that specific category without an accompanying reference, and thus, we deemed the category to have insufficient or weak evidence (giving it a “1”). Concurrently, if there were differing levels of evidence, we assigned the level of evidence that was found in the most recent systematic review for that category/subcategory.

The findings from each review were also cross-referenced with two expert bodies that have provided guidelines and/or most current evidence available for medical cannabis: (1) The United States’ National Academies of Science, Engineering and Medicine (NASEM)’s recent review of medical cannabis evidence [30] and (2) Canadian evidence for family practice recently compiled by Allan et al. [5].

## Results

Between April 2014 and January 2019, 65,245 adult patients were authorized for medical cannabis use following clinic-based medical assessments. Of these patients, 61,835 (94.8%) were from ON and 3410 (5.2%) were from AB. Across both provinces, the mean age of the patients was 52.8 ± 15.4 years and 53.9% of patients were female. The patients resided in neighborhoods distributed across all income quintiles—with the highest income quintile having the lowest proportion of patients (13.9%) (Table 1). The demographics of patients were very similar with respect to age (52.9 ± 15.4 vs. 51.8 ± 15.7 years) and sex (53.8% vs. 55.7% female) in ON and AB, respectively (Table 1). Both provinces also had similar distributions of self-reported questionnaire scores in the GAD-7, PHQ-9, and CAGE-AID assessments—with the majority of patients in both provinces self-reporting no or only mild issues with respect to anxiety (54.5%), depression (49.7%), or addiction (89.1%) (Table 1). Conversely, severe issues were reported by 25.7% of patients for anxiety and 12.5% for depression, and 10.9% reported problems with drugs and alcohol addiction.

**Table 1:** Characteristics of patients authorized medical cannabis in Ontario and Alberta, Canada (*n* = 65,245)

Characteristic	All patients (*N* = 65,245)	Ontario patients (*N* = 61,835)	Alberta patients (*N* = 3410)
*Age (years)*			
< 21	576 (0.9)	531 (0.9)	45 (1.3)
21–30	4732 (7.3)	4451 (7.2)	281 (8.2)
31–40	9814 (15.0)	9269 (15.0)	545 (16.0)
41–50	11,492 (17.6)	10,904 (17.6)	588 (17.2)
51–60	15,653 (24.0)	14,873 (24.1)	780 (22.9)
61–70	12,599 (19.3)	11,948 (19.3)	651 (19.1)
71–80	7012 (10.8)	6656 (10.8)	356 (10.4)
81–90	2960 (4.5)	2806 (4.5)	154 (4.5)
> 90	406 (0.6)	396 (0.6)	10 (0.3)
*Sex*			
Female	35,135 (53.9)	33,236 (53.8)	1899 (55.7)
Male	30,109 (46.1)	28,598 (46.2)	1511 (44.3)
Other	1 (0.0)	1 (0.0)	–
*Neighborhood income quintile*			
1	12,814 (19.6)	11,880 (19.2)	934 (27.4)
2	14,565 (22.3)	13,628 (22.0)	937 (27.5)
3	13,420 (20.6)	12,894 (20.9)	526 (15.4)
4	15,068 (23.1)	14,470 (23.4)	598 (17.5)
5	9063 (13.9)	8672 (14.0)	391 (11.5)
Missing	315 (0.5)	291 (0.5)	24 (0.7)
*GAD-7*	*N* = 37,303	*N* = 36,962	*N* = 341
None	11,809 (31.7)	11,701 (31.7)	108 (31.67)
Mild	8496 (22.8)	8419 (22.8)	77 (22.6)
Moderate	7400 (19.8)	7319 (19.8)	81 (23.8)
Severe	9598 (25.7)	9523 (25.8)	75 (22.0)
*PHQ-9*	*N* = 37,338	*N* = 36,995	*N* = 343
None	8777 (23.5)	8689 (23.5)	88 (25.7)
Mild	9769 (26.2)	9686 (26.2)	83 (24.2)
Moderate	8005 (21.4)	7912 (21.4)	93 (27.1)
Moderately severe	6106 (16.4)	6057 (16.4)	49 (14.3)
Severe	4681 (12.5)	4651 (12.6)	30 (8.8)
*CAGE*	*N* = 34,534	*N* = 34,316	*N* = 218
Negative	30,758 (89.1)	30,565 (89.1)	193 (88.5)
Positive	3776 (10.9)	3751 (10.9)	25 (11.5)
*Method of use*			
Smoking	33,024 (50.6)	31,301 (50.6)	1723 (50.5)
Vaping	30,044 (46.1)	29,536 (47.8)	508 (14.9)
Ingesting	42,366 (64.9)	40,448 (65.4)	1918 (56.3)
Topical use	2026 (3.1)	1980 (3.2)	46 (1.4)
Unknown	22,249 (34.1)	20,757 (33.6)	1492 (43.8)

Overall, 45,660 (70%) of the patients were categorized into more than one high-level condition (pain, mental health, autoimmune, cancer, sleep problems, neurological, gastrointestinal, other, or uncategorized). A total of 19,585 patients (30.0%) were in one category, 20,843 (32.0%) in two categories, 15,434 (23.7%) in three categories, and 9383 (14.4%) in four to eight categories. Both ON and AB patient demographics reflected the same top reasons for cannabis authorization: (1) pain (AB = 77%, ON = 76%), (2) mental health (AB = 32.9%, ON = 38.7%), and (3) sleep problems (AB = 28%, ON = 25%) (Table 2). Within the pain category, the primary complaints were due to chronic pain conditions resulting in musculoskeletal pain (25% of all patients, 33% of pain patients), arthritic pain (22.7% of all patients, 29.3% of pain patients), and neurologic pain (18.0% of all patients, 23.7% of pain patients). Patients seeking medical cannabis for mental health were mainly concerned with anxiety (24.7% of all patients, 64.1% of mental health patients) and depression (15.7% of all patients, 40.8% of mental health patients). Patients’ sleep problems were primarily insomnia (10.3% of all patients, 42.6% of sleep problem patients). Multiple sclerosis was another condition that was commonly cited (18.5% of all patients) as a reason for obtaining cannabis authorization; however, it is unclear whether the underlying reason may have been related to pain or spasticity concerns, or both.

**Table 2:** Reason for Medical Cannabis Authorization in Ontario and Alberta Adult Patients (*n* = 65,245)

Disorder	All (*N* = 65,245)	Ontario (*N* = 61,835)	Alberta (*N* = 3410)
*Pain*	49,621 (76.0)	46,987 (76.0)	2634 (77.2)
Endometriosis	444 (0.7)	416 (0.7)	28 (0.8)
Cancer pain	4933 (7.6)	4744 (7.7)	189 (5.5)
Arthritic pain	14,547 (22.7)	13,730 (22.2)	817 (24.0)
Neurologic pain	11,772 (18.0)	11,173 (18.1)	599 (17.6)
Musculoskeletal pain	16,451 (25.2)	15,607 (25.2)	1832 (53.7)
*Mental health*	25,081 (38.4)	23,960 (38.7)	1121 (32.9)
Anxiety	16,088 (24.7)	15,272 (24.7)	816 (23.9)
Depression	10,236 (15.7)	9727 (15.7)	509 (14.9)
PTSD	2581 (4.0)	2463 (4.0)	118 (3.5)
Bipolar	1340 (2.0)	1300 (2.1)	40 (1.2)
ADHD	984 (1.5)	935 (1.5)	49 (1.4)
Panic disorder	2331 (3.6)	2272 (3.7)	59 (1.7)
ADD	4854 (7.4)	4774 (7.7)	80 (2.4)
Mood disorder	2542 (3.9)	2457 (4.0)	85 (2.5)
Stress	2370 (3.6)	2285 (3.7)	85 (2.5)
OCD	424 (0.6)	398 (0.6)	26 (0.8)
Schizophrenia	325 (0.5)	319 (0.5)	6 (0.2)
*Autoimmune*	13,729 (21.0)	13,243 (21.4)	486 (14.2)
Multiple sclerosis	12,084 (18.5)	11,666 (18.9)	418 (12.3)
IBS	1741 (2.7)	1686 (2.7)	55 (1.6)
Lupus	301 (0.5)	285 (0.5)	16 (0.5)
Sjogren’s	97 (0.1)	90 (0.2)	7 (0.2)
*Sleep problems*	16,122 (24.7)	15,159 (24.5)	963 (28.2)
Insomnia	6706 (10.3)	6003 (9.7)	703 (20.6)
Fatigue	1835 (2.8)	1787 (2.9)	48 (1.4)
Sleep apnea	904 (1.4)	879 (1.4)	25 (0.7)
*Neurological*	5052 (7.7)	4829 (7.8)	223 (6.5)
Neuropathy	2079 (3.2)	1992 (3.2)	87 (2.6)
Parkinson’s	678 (1.0)	642 (1.0)	36 (1.1)
Seizure	1150 (1.8)	1111 (1.8)	39 (1.1)
Epilepsy	606 (0.9)	576 (0.9)	30 (0.9)
Restless leg syndrome	406 (0.6)	384 (0.6)	22 (0.7)
Tremor	894 (1.4)	850 (1.4)	44 (1.3)
ALS	108 (0.2)	107 (0.2)	1 (0.03)
Cerebral palsy	107 (0.2)	106 (0.2)	1 (0.03)
*Gastrointestinal*	2818 (4.3)	2710 (4.4)	108 (3.2)
Crohn’s	764 (1.2)	731 (1.2)	33 (1.0)
Colitis	469 (0.7)	444 (0.7)	25 (0.7)
*Other*	10,517 (16.1)	10,081 (16.3)	436 (12.8)
Osteoporosis	3274 (5.0)	3110 (5.0)	164 (4.8)
Nausea	2938 (4.5)	2855 (4.6)	83 (2.4)
Cancer-related nausea	1049 (1.6)	1025 (1.7)	24 (0.7)
Diabetes	1348 (2.1)	1251 (2.0)	97 (2.8)
Appetite	2307 (3.5)	2250 (3.6)	57 (1.7)
Cancer-related appetite	975 (1.5)	954 (1.5)	21 (0.6)
COPD	936 (1.4)	907 (1.5)	29 (0.8)
Concussion	524 (0.8)	501 (0.8)	23 (0.7)
Autism	198 (0.3)	188 (0.3)	10 (0.3)
Glaucoma	295 (0.4)	287 (0.5)	8 (0.2)
Huntington’s	17 (0.03)	16 (0.03)	1 (0.03)
*Uncategorized*	5796 (8.9)	5729 (9.3)	67 (2.0)

*PTSD* post-traumatic stress disorder, *ADHD* attention deficit hyperactivity disorder, *ADD* attention deficit disorder, *OCD* obsessive compulsive disorder, *IBS* irritable bowel syndrome, *ALS* amyotrophic lateral sclerosis, *COPD* chronic obstructive pulmonary disease

Of the conditions reported, those with substantial or conclusive evidence to support use include refractory pain related to cancer (7.6% of patients), chronic neuropathic pain (18.0% of patients), refractory spasticity conditions (7.7% of patients), short-term sleep improvement (24.7% of patients), and cancer-related nausea (1.6% of patients) [5, 23, 30]. While there is evidence to support the use for these conditions, it was noted as being weak to moderate evidence in the guidelines, so cannabis is not recommended as a first-line form of therapy. Glaucoma (0.4% of patients) was the only condition examined that had limited evidence, and reviews or guidelines suggested that cannabis should not be recommended for treatment of this condition [30]. There was no specific evidence to support or refute the use of medical cannabis for attention deficit disorder, obsessive compulsive disorder, lupus, Sjogren’s, or osteoporosis. The remaining conditions examined (8.9%) had either limited or insufficient evidence available, so recommendations could not be made.

In all, roughly 16,483 (25.3%) patients sought medical cannabis for reasons with limited or insufficient evidence to support the therapeutic benefits of medical cannabis for their condition based on currently available evidence (Additional file 1: Appendix A). This includes some of the conditions most frequently cited as reasons for prescription such as non-neuropathic musculoskeletal pain (25.2% of patients), arthritic pain (22.7% of patients), anxiety (24.7% of patients), and depression (15.7% of patients). The majority of the remaining conditions, as mentioned, have either no available evidence or show weak evidence in showing an association with its use for that particular condition.

## Discussion

This population-based cohort study showed important characteristics about adult patients who were medically authorized for cannabis in ON and AB, Canada. Overall, the demographics of the adult population in ON and AB were comparable for both mean age and sex at the index date of authorization. The primary reasons for cannabis authorization were similar between ON and AB with pain, mental health, and sleep problems as the most common.

For the other 70% of patients in ON and AB, they were categorized into more than one high-level condition (pain, mental health, autoimmune, cancer, sleep problems, neurological, gastrointestinal, other, or uncategorized) for cannabis authorization, suggesting these patients have multiple comorbidities which are not currently being adequately addressed with more traditional medical therapies. This also may suggest that the nature of therapeutic applications of cannabis is multifaceted. For example, a patient experiencing cancer pain may also suffer from sleep problems. Hence, the multi-symptom [16] nature of patients who seek cannabis may make identification of the efficacy of medical cannabis, per symptom, difficult. From a quality of life standpoint, medical cannabis may be viewed as effective for one symptom (ex: sleep)—however, if improvement of pain or cancer were also included as outcomes, then cannabis may appear ineffective. These outcomes may be a contributing factor to the mixed results [2] on cannabis efficacy in the literature.

Previous research studies have reported a level of substantial evidence for medical cannabis’ use in the alleviation of pain [5, 27, 29]. Both Canadian clinicians [5] and the NASEM [30] have concurred that medical cannabis use may be effective in the management of chronic pain. In addition, they also suggest medical cannabis may be effective for chemotherapy-induced vomiting/nausea, and multiple sclerosis spasticity symptoms [5, 30]. Further, previous research has shown that there is an inherent psychological [31] element to medical cannabis use, in particular for relieving anxiety [4, 14] and depression [18, 19]. A significant portion of mental health outcome studies on medical cannabis also included its frequent utilization for both sleep problems [21, 22] and post-traumatic stress disorder [13, 15]. Otherwise, clinician recommendations emphasize limiting medical cannabis use for any other ailment (as a first-choice treatment plan) [5, 23].

Interestingly, this study shows that AB and ON physicians are prescribing medical cannabis for over 50 listed conditions (Additional file 1: Table S1). At least 25% of patients in this study were using medical cannabis for reasons not currently supported by evidence. Moreover, 25% is likely a very conservative estimate as it assumed that all reported reasons met the specific criteria which has the support of evidence (i.e., all cancer pain was refractory cancer pain, all patients stating diabetes and pain had neuropathic diabetic pain, all sleep problems were short term, etc.). This observation aligns with the systematic review by Lim et al. [12]—that shows that a significant portion of medical cannabis is used for a diverse range of specific health conditions or disorders. Furthermore, the systematic review showed little to no evidence supporting medical cannabis use on the treatment of these other health conditions. Likewise, NASEM [23] has also reported that there is limited to insufficient evidence for medical cannabis use on any health conditions other than chronic pain. This is also supported by Canadian evidence [5] on avoiding cannabis as a first line or second line of treatment. The guidelines, however, do make an exception for a small consideration of neuropathic pain—but this is emphasized as a weak recommendation.

Although limited evidence existed within reviews and guidelines for many of the conditions, that is not to suggest patients may not be experiencing benefit from medical cannabis for their symptoms of the condition but that the scientific evidence to support the effectiveness and association of medical cannabis with the health condition has not been established; or in many cases, research has not been conducted at all. However, it could also speak to potential lack of education available in regard to medical cannabis among physicians as recent studies have reported that Canadian physicians and physicians-in-training desire more education and knowledge in regard to the protocol and treatment plans for medical cannabis therapy [32]. Indeed, one of the critical gaps in their training was reported to be understanding how to implement effective medical cannabis treatment plans that accounted for all risks and benefits of medical cannabis [33]. Conversely, other physicians [34] have also reported hesitancy or complete reluctance to authorize medical cannabis as a result of this lack of guidance and education. Thus, our study provides ongoing surveillance for understanding physician prescribing behavior and potential educational needs on the evidence of medical cannabis for these certain types of health conditions.

It is important to consider that, although the use of medical cannabis for certain conditions may not be supported by current reviews or guidelines, the use of cannabis through the medical system (i.e., prescribed by physicians or nurse practitioners), as opposed to self-medication through non-medical retail sales which are now legal in Canada, may have important implications for harm reduction. Indeed, inclusion of physicians and other health professionals that have a full understanding of the patients comorbidities and other medication use would be expected to identify and reduce potential adverse effects of cannabis as patients can be actively monitored and assessed by qualified professionals. Moreover, if medical cannabis is felt to improve symptoms of a condition, there may be potential additional harm reductions with its use. For example, pain was one of the highest conditions noted where medical cannabis is being used in this cohort of patients. We have previously shown that cannabis may have important impacts to help reduce the use of opioids for chronic pain at the population level [35]. Thus, medical cannabis authorization could be a potential strategy to minimize harm associated with opioid use.

The major strength of this study is that it is currently Canada’s largest study on medical cannabis authorized adult patients. Therefore, the data from this study provide current and critical information about Canadian patients seeking cannabis as a form of therapy for various health reasons. The findings from this study contribute to the knowledge base on population-level use beyond its non-medical uses. Our analyses further provide evidence on important subgroups of patients who are currently using medical cannabis for treatment of over 200 conditions.

This study relied on previously published reviews or guidelines in the assessment of evidence. It is possible that other evidence exists for the use of medical cannabis within certain conditions that were not addressed in the previous reviews or guidelines and therefore would not have been included in our levels of evidence assessments. Another limitation to this study is that our data are restricted to only patients who have attended a cannabis medical clinic. It is also possible that responders to the questionnaires provide information overstating their health symptoms as a means to increase their likelihood of being authorized medical cannabis. We also did not appraise the level of evidence ourselves and used the appraisals from other studies (Additional file 1: Appendix A). The Canadian evidence [5] and the NASEM [23] guidelines often had differing opinions on the strength of evidence with the US guidelines saying substantive evidence and the Canadian evidence reporting weak evidence. When differences were found, the recommendations from the Canadian evidence recommendations were used. Finally, we have no method to determine whether cannabis was being considered as a first, second, or alterative line of therapy—in most cases it is more than plausible it was not used as first line.

## Conclusions

Overall, medical cannabis continues to be authorized for numerous conditions that are not supported by current guidelines or reviews. Although evidence gaps may exist, the inclusion of clinicians in the prescribing and monitoring of medical cannabis’ effects on patients is expected to have important implications for harm reduction for the use of cannabis in these conditions. Moreover, evidence generated from patients and front-line clinicians prescribing cannabis will contribute to the evidence base and will help focus epidemiological and randomized controlled trials on medical cannabis use in these health conditions to identify its safety and health benefits at the population level. We believe our findings contribute ongoing data on medical cannabis authorization to frontline clinicians, healthcare administration, and federal/provincial governments to capture clarity on the current population of medical cannabis users, reasons for authorization, and the paucity of evidence to help guide evidence-based decisions.

A key future direction for this cohort, and others, will be to evaluate the impacts and safety of medical cannabis over both the short and long terms for the vast array of conditions noted. Notably, this study is not meant to correlate high frequency of authorization as recommendation for its usage. Adults seeking medical cannabis should continue to approach this medication with caution and ideally under the direct supervision of qualified health professionals to help reduce any harms associated with its use.

## Supplementary Information

**Additional file 1: Supplemental Table 1**. Keywords used to code the reason for medical cannabis authorization; **Appendix A**. List of Literature for the Evidence Appraisal.

## Data Availability

The dissemination of data results to study participants and or patient organizations in this research project is not possible/applicable. This study is based in part on data provided by Canadian Cannabis Clinics and CanvasRx Inc. The interpretation and conclusions contained herein are those of the researchers and do not necessarily represent the views of Canadian Cannabis Clinics or Canvas Rx Inc., each of whom do not express any opinion in relation to this study.

## Abbreviations

AB: Alberta
CAGE-AID: CAGE Questionnaire Adapted to Include Drugs
NASEM: National Academies of Science, Engineering and Medicine
ON: Ontario
GAD-7: Generalized Anxiety Disorder 7-item
PHQ-9: Patient Health Questionnaire

## References

[1] Ko GD, Bober SL, Mindra S, Moreau JM (2016). Medical cannabis—the Canadian perspective. J Pain Res.

[2] Whiting PF, Wolff RF, Deshpande S, Di Nisio M, Duffy S, Hernandez AV (2015). Cannabinoids for medical use: a systematic review and meta-analysis. JAMA.

[3] Makary P, Parmar JR, Mims N, Khanfar NM, Freeman RA (2018). Patient counseling guidelines for the use of cannabis for the treatment of chemotherapy-induced nausea/vomiting and chronic pain. J Pain Palliat Care Pharmacother.

[4] Turna J, Patterson B, Van Ameringen M (2017). Is cannabis treatment for anxiety, mood, and related disorders ready for prime time?. Depress Anxiety.

[5] Allan GM, Ramji J, Perry D, Ton J, Beahm NP, Crisp N (2018). Simplified guideline for prescribing medical cannabinoids in primary care. Can Fam Phys.

[6] Lake S, Kerr T, Montaner J (2015). Prescribing medical cannabis in Canada: Are we being too cautious?. Can J Public Health.

[7] Tafelski S, Hauser W, Schafer M (2016). Efficacy, tolerability, and safety of cannabinoids for chemotherapy-induced nausea and vomiting—a systematic review of systematic reviews. Schmerz.

[8] Wall MM, Liu J, Hasin DS, Blanco C, Olfson M (2019). Use of marijuana exclusively for medical purposes. Drug Alcohol Depend.

[9] Blake A, Wan BA, Malek L, DeAngelis C, Diaz P, Lao N (2017). A selective review of medical cannabis in cancer pain management. Ann Palliat Med.

[10] Maharajan MK, Yong YJ, Yip HY, Woon SS, Yeap KM, Yap KY (2019). Medical cannabis for chronic pain: can it make a difference in pain management?. J Anesth.

[11] Campbell G, Hall WD, Peacock A, Lintzeris N, Bruno R, Larance B (2018). Effect of cannabis use in people with chronic non-cancer pain prescribed opioids: findings from a 4-year prospective cohort study. Lancet Public Health.

[12] Lim K, See YM, Lee J (2017). A systematic review of the effectiveness of medical cannabis for psychiatric, movement and neurodegenerative disorders. Clin Psychopharmacol Neurosci.

[13] Steenkamp MM, Blessing EM, Galatzer-Levy IR, Hollahan LC, Anderson WT (2017). Marijuana and other cannabinoids as a treatment for posttraumatic stress disorder: a literature review. Depress Anxiety.

[14] Sarris J, Sinclair J, Karamacoska D, Davidson M, Firth J (2020). Medicinal cannabis for psychiatric disorders: a clinically-focused systematic review. BMC Psychiatry.

[15] Orsolini L, Chiappini S, Volpe U, Berardis D, Latini R, Papanti GD (2019). Use of medicinal cannabis and synthetic cannabinoids in post-traumatic stress disorder (PTSD): a systematic review. Medicina.

[16] Turna J, Simpson W, Patterson B, Lucas P, Van Ameringen M (2019). Cannabis use behaviors and prevalence of anxiety and depressive symptoms in a cohort of Canadian medicinal cannabis users. J Psychiatr Res.

[17] Shannon S, Lewis N, Lee H, Hughes S (2019). Cannabidiol in anxiety and sleep: a large case series. Perm J.

[18] Feingold D, Brill S, Goor-Aryeh I, Delayahu Y, Lev-Ran S (2018). The association between severity of depression and prescription opioid misuse among chronic pain patients with and without anxiety: a cross-sectional study. J Affect Disord.

[19] Taub S, Feingold D, Rehm J, Lev-Ran S (2018). Patterns of cannabis use and clinical correlates among individuals with major depressive disorder and bipolar disorder. Compr Psychiatry.

[20] Katz D, Katz I, Porat-Katz BS, Shoenfeld Y (2017). Medical cannabis: Another piece in the mosaic of autoimmunity?. Clin Pharmacol Ther.

[21] Sznitman SR, Vulfsons S, Meiri D, Weinstein G (2020). Medical cannabis and insomnia in older adults with chronic pain: a cross-sectional study. BMJ Support Palliat Care.

[22] Choi S, Huang BC, Gamaldo CE (2020). Therapeutic uses of cannabis on sleep disorders and related conditions. J Clin Neurophysiol.

[23] Abrams DI (2018). The therapeutic effects of cannabis and cannabinoids: an update from the National Academies of Sciences, Engineering and Medicine report. Eur J Intern Med.

[24] Andrews CN, Devlin SM, Le Foll B, Fischer B, Tse F, Storr M (2019). Canadian association of gastroenterology position statement: use of cannabis in gastroenterological and hepatic disorders. J Can Assoc Gastroenterol.

[25] Agarwal R, Burke SL, Maddux M (2019). Current state of evidence of cannabis utilization for treatment of autism spectrum disorders. BMC Psychiatry.

[26] Abdallah SJ, Smith BM, Ware MA, Moore M, Li PZ, Bourbeau J (2018). Effect of vaporized cannabis on exertional breathlessness and exercise endurance in advanced chronic obstructive pulmonary disease. A randomized controlled trial. Ann Am Thorac Soc.

[27] O'Connell M, Sandgren M, Frantzen L, Bower E, Erickson B (2019). Medical cannabis: effects on opioid and benzodiazepine requirements for pain control. Ann Pharmacother.

[28] Allan GM, Finley CR, Ton J, Perry D, Ramji J, Crawford K (2018). Systematic review of systematic reviews for medical cannabinoids: pain, nausea and vomiting, spasticity, and harms. Can Fam Phys.

[29] Romero-Sandoval EA, Fincham JE, Kolano AL, Sharpe BN, Alvarado-Vazquez PA (2018). Cannabis for chronic pain: challenges and considerations. Pharmacotherapy.

[30] The Health Effects of Cannabis and Cannabinoids: The Current State of Evidence and Recommendations for Research. The National Academies Collection: Reports funded by National Institutes of Health. Washington, DC; 2017. 28182367

[31] Botsford SL, Yang S, George TP (2020). Cannabis and cannabinoids in mood and anxiety disorders: impact on illness onset and course, and assessment of therapeutic potential. Am J Addict.

[32] St Pierre M, Matthews L, Walsh Z (2020). Cannabis education needs assessment among Canadian physicians-in-training. Complement Ther Med.

[33] Ziemianski D, Capler R, Tekanoff R, Lacasse A, Luconi F, Ware MA (2015). Cannabis in medicine: a national educational needs assessment among Canadian physicians. BMC Med Educ.

[34] Ng JY, Gilotra K, Usman S, Chang Y, Busse JW (2021). Attitudes toward medical cannabis among family physicians practising in Ontario, Canada: a qualitative research study. CMAJ Open.

[35] Lee C, Lin M, Martins KJB, Dyck JRB, Klarenbach S, Richer L (2021). Opioid use in medical cannabis authorization adult patients from 2013 to 2018: Alberta, Canada. BMC Public Health.

