# Supplementary material for: Gaps in evidence for the use of medically authorized cannabis: Ontario and Alberta, Canada
Source: Harm Reduct J. 2021 Jun 8;18:61. doi: 10.1186/s12954-021-00509-0 (PMC8186125; doi:10.1186/s12954-021-00509-0)
Supplement: Supplementary file 1 — Additional file 1: Supplemental Table 1. Keywords used to code the reason for medical cannabis authorization; Appendix A. List of Literature for the Evidence Appraisal. [file 12954_2021_509_MOESM1_ESM.docx]

**Supplemental Table 1. Keywords used to code the reason for medical cannabis authorization.**

| **Disorder** | **Keywords** |
| --- | --- |
| Pain | pain, injury, ache, strain, and any of the below  categories |
| Endometriosis | endometriosis |
| Cancer Pain | Any of Pain (see above) AND (cancer, ca , metastatic, malignant, tumour, tumor, carcinoma, lymphoma, melanoma, glioblastoma, myeloma, myelodys, leukemia, OR sarcoma) |
| Arthritic Pain | Any of Pain (see above) AND ( oa , arthrit, arthral, osteoarthrit, rheumatoid, ra , ankylosing spond, OR ankylosis spond) |
| Neurologic Pain | (pain OR injury AND nerve OR neuropathic), radiculopa, sciatica, neuralgia, fibro, migraine, migrane, headache, carpal tunnel, carpel tunnel, OR spina bif |
| Musculoskeletal Pain | (pain Or injury AND back, lumbar, neck, knee, shoulder, hip, leg, feet, foot, abdominal, arm, myofascial, ankle, pelvic), lbp, cbp, ddd, degenerative disc, spinal stenosis, scoliosis, herniated dis, disc herniation, spondylo, spondylitis, rotator cuff, mva, mvc, fracture, tendon, tendin, spasm, OR myalgia |
| Mental Health | Any of the below categories |
| Anxiety | Anxiety, gad |
| Depression | Depression, depressive, mdd |
| PTSD | Post traumatic stress disorder, ptsd |
| Bipolar | Bipolar, bi-polar |
| ADHD | adhd, attention deficit hyperactivity |
| Panic Disorder | Panic |
| ADD | add, attention deficit disorder |
| Mood Disorder | Mood |
| Stress | stress |
| OCD | ocd, obsessive compulsive |
| Schizophrenia | schizo |
| Autoimmune | Any of the below categories |
| Multiple Sclerosis | ms, multiple sclerosis |
| IBS | ibs, irritable bowel |
| Lupus | lupus |
| Sjogren’s | sjogren |
| Sleep problems | Sleep, or any of the below |
| Insomnia | insomnia |
| Fatigue | fatigue |
| Sleep Apnea | sleep apnea |
| Neurological | Any of the below categoreis |
| Neuropathy | neuropathy |
| Parkinson’s | parkinson |
| Seizure | seizure |
| Epilepsy | epilepsy |
| Restless Leg Syndrome | restless leg |
| Tremor | tremor |
| ALS | als |
| Cerebral Palsy | cerebral palsy |
| Gastrointestinal | IBS or any of the below |
| Crohn’s | crohn, chrons, chron's, chrohn |
| Colitis | colitis |
| Other | Any of the below categories |
| Osteoporosis | osteo |
| Nausea | nausea |
| Cancer Related Nausea | Nausea AND (cancer, ca , metastatic, malignant, tumour, tumor, carcinoma, lymphoma, melanoma, glioblastoma, myeloma, myelodys, leukemia, OR sarcoma) |
| Diabetes | diabet |
| Appetite | appetite |
| Cancer Related Appetite | Appetite AND (cancer, ca , metastatic, malignant, tumour, tumor, carcinoma, lymphoma, melanoma, glioblastoma, myeloma, myelodys, leukemia, OR sarcoma) |
| COPD | copd |
| Concussion | concussion |
| Autism | autism, autistic, asperger |
| Glaucoma | glaucoma |
| Huntington’s | huntington |

*PTSD:* Post-traumatic stress disorder *ADHD*: Attention deficit hyperactivity disorder *ADD*: Attention deficit disorder *OCD*: Obsessive compulsive disorder *IBS*: Irritable bowel syndrome *ALS*: Amyotrophic lateral sclerosis *COPD*: Chronic obstructive pulmonary disease

**Appendix A: List of Literature for the Evidence Appraisal**

| **Disorder** | **Canadian^1^ Recommendations** | **NASEM^2^** | **Other Reviews** |
| --- | --- | --- | --- |
| Pain  Acute pain  Chronic pain^3-5^ | 1  1 | 2  3 | 2  2 |
|  |  |  |  |
| Cancer Pain^6-8^ | 2 | 3 | 2 |
| Arthritic Pain^9-11^ | 1 | 1 | 1 |
| Neurologic Pain^12^ | 1 | 2 | 2 |
| Musculoskeletal Pain^13,14^ | 1 | 1 | 1 |
| Mental Health^15-17^ |  |  |  |
| Anxiety^18^ | 1 | 2 | 2 |
| Depression^19^ | 1 | 1 | 1 |
| PTSD^20-22^ | 1 | 1 | 2 |
| Bipolar^23^ | 1 | 2 | 1 |
| ADHD | 1 | 1 | 2 |
| Panic Disorder | 1 | 1 | 1 |
| ADD | 1 | 1 | 1 |
| Mood Disorder^24^ | 1 | 1 | 1 |
| Stress | 1 | 2 | 1 |
| OCD | 1 | 1 | 1 |
| Schizophrenia^25,26^ | 1 | 1 | 2 |
| Autoimmune |  |  |  |
| Multiple Sclerosis^27^ | 1 | 2 | 2 |
| IBS^28^ | 1 | 1 | 1 |
| Lupus^29^ | 1 | 1 | 1 |
| Sjogren’s | 1 | 1 | 1 |
| Sleep problems^30-32^ |  |  |  |
| Insomnia | 1 | 2 | 2 |
| Fatigue | 1 | 1 | 1 |
| Sleep Apnea | 1 | 2 | 2 |
| Neurological^33,34^ | 1 |  |  |
| Neuropathy | 1 | 1 | 1 |
| Parkinson’s^35^ | 1 | 1 | 1 |
| Seizure^36^ | 1 | 1 | 2 |
| Epilepsy^37^ | 1 | 1 | 1 |
| Restless Leg Syndrome | 1 | 1 | 1 |
| Tremor | 1 | 1 | 1 |
| ALS | 1 | 1 | 1 |
| Cerebral Palsy | 1 | 1 | 1 |
| Gastrointestinal^38^ |  |  |  |
| Crohn’s^39^ | 1 | 1 | 1 |
| Colitis^39^ | 1 | 1 | 1 |
| Other |  |  |  |
| Osteoporosis | 1 | 1 | 1 |
| Nausea | 2 | 3 | 2 |
| Cancer Related Nausea | 2 | 3 | 2 |
| Diabetes | 1 | 1 | 1 |
| Appetite | 1 | 1 | 1 |
| Cancer Related Appetite^40^ | 1 | 2 | 1 |
| COPD | 1 | 1 | 1 |
| Concussion | 1 | 1 | 1 |
| Autism | 1 | 1 | 1 |
| Glaucoma | 1 | 1 | 1 |
| Huntington’s^41^ | 1 | 1 | 1 |

**Legend:**

1 – None or weak evidence for efficacy

2 – Limited or moderate evidence for efficacy

3 – Substantive or conclusive evidence

**References**

1. Allan GM, Finley CR, Ton J, et al. Systematic review of systematic reviews for medical cannabinoids: Pain, nausea and vomiting, spasticity, and harms. *Can Fam Physician.* 2018;64(2):e78-e94.

2. In: *The Health Effects of Cannabis and Cannabinoids: The Current State of Evidence and Recommendations for Research.* Washington (DC)2017.

3. Mucke M, Phillips T, Radbruch L, Petzke F, Hauser W. Cannabis-based medicines for chronic neuropathic pain in adults. *Cochrane Database Syst Rev.* 2018;3:CD012182.

4. Okusanya BO, Asaolu IO, Ehiri JE, Kimaru LJ, Okechukwu A, Rosales C. Medical cannabis for the reduction of opioid dosage in the treatment of non-cancer chronic pain: a systematic review. *Syst Rev.* 2020;9(1):167.

5. Whiting PF, Wolff RF, Deshpande S, et al. Cannabinoids for Medical Use: A Systematic Review and Meta-analysis. *JAMA.* 2015;313(24):2456-2473.

6. Boland EG, Bennett MI, Allgar V, Boland JW. Cannabinoids for adult cancer-related pain: systematic review and meta-analysis. *BMJ Support Palliat Care.* 2020;10(1):14-24.

7. Hauser W, Welsch P, Klose P, Radbruch L, Fitzcharles MA. Efficacy, tolerability and safety of cannabis-based medicines for cancer pain : A systematic review with meta-analysis of randomised controlled trials. *Schmerz.* 2019;33(5):424-436.

8. Wright P, Walsh Z, Margolese S, et al. Canadian clinical practice guidelines for the use of plant-based cannabis and cannabinoid-based products in the management of chronic non-cancer pain and co-occurring conditions: protocol for a systematic literature review. *BMJ Open.* 2020;10(5):e036114.

9. Stockings E, Campbell G, Hall WD, et al. Cannabis and cannabinoids for the treatment of people with chronic noncancer pain conditions: a systematic review and meta-analysis of controlled and observational studies. *Pain.* 2018;159(10):1932-1954.

10. Berg MVD, John M, Black M, et al. Cannabis-based medicinal products in arthritis, a painful conundrum. *N Z Med J.* 2020;133(1515):35-45.

11. Madden K, George A, van der Hoek NJ, Borim FM, Mammen G, Bhandari M. Cannabis for pain in orthopedics: a systematic review focusing on study methodology. *Can J Surg.* 2019;62(6):369-380.

12. Aviram J, Samuelly-Leichtag G. Efficacy of Cannabis-Based Medicines for Pain Management: A Systematic Review and Meta-Analysis of Randomized Controlled Trials. *Pain Physician.* 2017;20(6):E755-E796.

13. Johal H, Devji T, Chang Y, Simone J, Vannabouathong C, Bhandari M. Cannabinoids in Chronic Non-Cancer Pain: A Systematic Review and Meta-Analysis. *Clin Med Insights Arthritis Musculoskelet Disord.* 2020;13:1179544120906461.

14. Cameron EC, Hemingway SL. Cannabinoids for fibromyalgia pain: a critical review of recent studies (2015-2019). *J Cannabis Res.* 2020;2(1):19.

15. Bonaccorso S, Ricciardi A, Zangani C, Chiappini S, Schifano F. Cannabidiol (CBD) use in psychiatric disorders: A systematic review. *Neurotoxicology.* 2019;74:282-298.

16. Khan R, Naveed S, Mian N, Fida A, Raafey MA, Aedma KK. The therapeutic role of Cannabidiol in mental health: a systematic review. *J Cannabis Res.* 2020;2(1):2.

17. Sarris J, Sinclair J, Karamacoska D, Davidson M, Firth J. Medicinal cannabis for psychiatric disorders: a clinically-focused systematic review. *BMC Psychiatry.* 2020;20(1):24.

18. Kosiba JD, Maisto SA, Ditre JW. Patient-reported use of medical cannabis for pain, anxiety, and depression symptoms: Systematic review and meta-analysis. *Soc Sci Med.* 2019;233:181-192.

19. Lev-Ran S, Roerecke M, Le Foll B, George TP, McKenzie K, Rehm J. The association between cannabis use and depression: a systematic review and meta-analysis of longitudinal studies. *Psychol Med.* 2014;44(4):797-810.

20. Orsolini L, Chiappini S, Volpe U, et al. Use of Medicinal Cannabis and Synthetic Cannabinoids in Post-Traumatic Stress Disorder (PTSD): A Systematic Review. *Medicina (Kaunas).* 2019;55(9).

21. O'Neil ME, Nugent SM, Morasco BJ, et al. Benefits and Harms of Plant-Based Cannabis for Posttraumatic Stress Disorder: A Systematic Review. *Ann Intern Med.* 2017;167(5):332-340.

22. Hindocha C, Cousijn J, Rall M, Bloomfield MAP. The Effectiveness of Cannabinoids in the Treatment of Posttraumatic Stress Disorder (PTSD): A Systematic Review. *J Dual Diagn.* 2020;16(1):120-139.

23. Jordan Walter T, Pocuca N, Young JW, Geyer MA, Minassian A, Perry W. The relationship between cannabis use and cognition in people with bipolar disorder: A systematic scoping review. *Psychiatry Res.* 2020;297:113695.

24. Mammen G, Rueda S, Roerecke M, Bonato S, Lev-Ran S, Rehm J. Association of Cannabis With Long-Term Clinical Symptoms in Anxiety and Mood Disorders: A Systematic Review of Prospective Studies. *J Clin Psychiatry.* 2018;79(4).

25. Ghabrash MF, Coronado-Montoya S, Aoun J, et al. Cannabidiol for the treatment of psychosis among patients with schizophrenia and other primary psychotic disorders: A systematic review with a risk of bias assessment. *Psychiatry Res.* 2020;286:112890.

26. Patel S, Khan S, M S, Hamid P. The Association Between Cannabis Use and Schizophrenia: Causative or Curative? A Systematic Review. *Cureus.* 2020;12(7):e9309.

27. Nielsen S, Germanos R, Weier M, et al. The Use of Cannabis and Cannabinoids in Treating Symptoms of Multiple Sclerosis: a Systematic Review of Reviews. *Curr Neurol Neurosci Rep.* 2018;18(2):8.

28. Volz MS, Siegmund B, Hauser W. [Efficacy, tolerability, and safety of cannabinoids in gastroenterology: A systematic review]. *Schmerz.* 2016;30(1):37-46.

29. Guillouard M, Authier N, Pereira B, Soubrier M, Mathieu S. Cannabis use assessment and its impact on pain in rheumatologic diseases: a systematic review and meta-analysis. *Rheumatology (Oxford).* 2020.

30. Suraev AS, Marshall NS, Vandrey R, et al. Cannabinoid therapies in the management of sleep disorders: A systematic review of preclinical and clinical studies. *Sleep Med Rev.* 2020;53:101339.

31. Bhagavan C, Kung S, Doppen M, et al. Cannabinoids in the Treatment of Insomnia Disorder: A Systematic Review and Meta-Analysis. *CNS Drugs.* 2020;34(12):1217-1228.

32. Gates PJ, Albertella L, Copeland J. The effects of cannabinoid administration on sleep: a systematic review of human studies. *Sleep Med Rev.* 2014;18(6):477-487.

33. Lim K, See YM, Lee J. A Systematic Review of the Effectiveness of Medical Cannabis for Psychiatric, Movement and Neurodegenerative Disorders. *Clin Psychopharmacol Neurosci.* 2017;15(4):301-312.

34. Scott EP, Brennan E, Benitez A. A Systematic Review of the Neurocognitive Effects of Cannabis Use in Older Adults. *Curr Addict Rep.* 2019;6(4):443-455.

35. Bougea A, Koros C, Simitsi AM, Chrysovitsanou C, Leonardos A, Stefanis L. Medical cannabis as an alternative therapeutics for Parkinsons' disease: Systematic review. *Complement Ther Clin Pract.* 2020;39:101154.

36. Lattanzi S, Brigo F, Trinka E, et al. Efficacy and Safety of Cannabidiol in Epilepsy: A Systematic Review and Meta-Analysis. *Drugs.* 2018;78(17):1791-1804.

37. Elliott J, DeJean D, Clifford T, et al. Cannabis-based products for pediatric epilepsy: A systematic review. *Epilepsia.* 2019;60(1):6-19.

38. Langhorst J, Wulfert H, Lauche R, et al. Systematic review of complementary and alternative medicine treatments in inflammatory bowel diseases. *J Crohns Colitis.* 2015;9(1):86-106.

39. Kafil TS, Nguyen TM, MacDonald JK, Chande N. Cannabis for the treatment of Crohn's disease. *Cochrane Database Syst Rev.* 2018;11:CD012853.

40. Smith LA, Azariah F, Lavender VT, Stoner NS, Bettiol S. Cannabinoids for nausea and vomiting in adults with cancer receiving chemotherapy. *Cochrane Database Syst Rev.* 2015(11):CD009464.

41. Akinyemi E, Randhawa G, Longoria V, Zeine R. Medical Marijuana Effects in Movement Disorders, Focus on Huntington Disease; A Literature Review. *J Pharm Pharm Sci.* 2020;23.
